# Annotations

**Published:** 1890-05-03

**Authors:** 


### Annotations.

**Degraissage.**

New operations in surgery are of comparatively frequent occurrence. They seem to surprise the public a good deal sometimes, though medical men of philosophic mind and adequate physiological knowledge often anticipate them by long periods. The opening of the chest and the removal of diseased portions of lung has long been looked for by rational practitioners. The actual performance of an operation of this kind caused quite a sensation a short time ago in the non-medical world. The last new operation, that of making a fat man lean, has not perhaps been so generally expected by doctors. To many it will no doubt come as a surprise, though no medical man will look upon it as at all wonderful. Compared with such operations as ovariotomy it is mere child's play. Whoever has removed a fatty tumour will probably look upon *degraissage* as a mere extension of accepted practice. Still the idea of making a fat man lean is somewhat novel; and if the practice is not carried to excess it may be considered a small advance in general surgery. A contemporary states that Dr. Marx and Dr. Demars have cut away more than four pounds of fat from M. Hiroguelle, a literary man. The doctors, and all Paris with them, seem to be very proud of the feat, and M. Hiroguelle is charmed. It is now more than a week since the operation was performed, and the patient, besides being delighted with the improvement in his figure, says he feels quite well—in fact, with the exception of a headache from the chloroform he has felt no bad effects at all. No wonder he is looking forward with quite cheerful spirits to the "paring" away of other adipose humps and prominences that mar his masculine beauty. But it is not to be forgotten that there are such things as "resurrections" in the world. Drs. Marx and Demars may skilfully cut away super-abundant fat; but have they got the repressive talisman that will prevent new fat from growing in the place of the old? That seems to be the most important question raised by the new departure. Thousands of people will be glad to improve their figures and to lighten the adipose burdens of life. But will the improvement be permanent? That is the question. The truth can only be slowly demonstrated by lapse of time. M. Hiroguelle, it is to be hoped, will remain the hero of the hour for at least a year or two. We do not counsel English surgeons to try the operation until a sufficient leng th of time has elapsed for the results on the Frenchman to be seen. There is, not after all, any very great merit or general advantage in fat removal. Such honour as there is will of course be claimed by French surgeons, and, so far as we are concerned, they are very welcome to it.

**Hops and their Rivals.**

Brewing is one of the principal industries of this country, and beer one of the chief articles of diet. Whether a man agree with beer drinking or not, he cannot deny that so long as people do drink beer, it is very desirable that the beer shall be honest and good. From early times in English history, the country has been famous for its beer; and until quite recent years, there was never any doubt on the point that ordinary beer was a more or less skilful decoction of malt and hops. In some parts of England, Yorkshire for example, every housewife, so recently as thirty years ago, knew how to brew, and every house had its brewing day. Excellent beer some of the cottagers made, whilst the larger houses had their "Brown October," which could be kept for years, and was warranted to make a man very happy and very drunk within the briefest possible space of time. Those days, that seem to us now so honest and so remote, knew nothing of substitutes for hops. Other fermented liquors were made and used, but they were never called by the unqualified name of "beer." They were described as "spruce beer," "ginger" beer, "nettle" beer, and even "treacle" beer, and the like. The word "beer" no longer connotes a simple fermented decoction of malt and hops. According to evidence given the other day before the Select Committee of the House of Commons on the Hop Industry, beer may mean malt and calumba, malt and camomile, malt and quassia, malt and guinea-green, malt and chiretta, and a good many other things. This is not honest, and it is not English. It is, in plain words, down-right knavery. Of course, as every doctor knows, calumba, camomile, quassia, and chiretta, are not merely harmless, but have a certain tonic value. They do not, however, possess either the same value or the same flavour as hops; and even if they did, it is still a swindling trick to substitute them for hops, without acknowledgment, and to sell the liquids produced from their decoctions as *bona fide* beers. Some of the witnesses before the Commission contended that beers brewed from other things than hops should be described according to their actual quality. Instead of being called simply "beer" the quassia brew should be called "quassia beer," the camomile brew "camomile beer," the chiretta brew "chiretta beer," and so on. That this is what ought to be done, even offending brewers may easily be convinced by a simple illustration. They sell their quassia and calumba mixtures as "beer," because the general taste of the liquids covers up and hides the special taste of the quassia, calumba, etc.; and they rely upon the drinker not finding out the deception. But supposing they were to buy of the hop merchant five hundred-weights of hops according to a particular sample, and they were to find on delivery that two of the five hundred-weight were quassia or calumba and not hops at all, what would they say to the hop merchant? They would return the quassia immediately, and demand either the full weight of hops instead, or the return of their money. If the hop merchant made a difficulty they would not only let loose the law upon him, but would boycott both him and his warehouse for all future time. Yet that which would make them so angry if done to themselves by the hop merchant, is exactly what they are constantly doing to their customers when they use quassia, calumba, chiretta, and the like as entire or partial substitutes for hops. If they were honest men they would describe their beers according to the actual contents of the barrels. But since it is only too evident that they are not honest, though they may be wealthy, Government should at once charge itself with their conversion, and compel them under adequate penalties to act fairly and squarely.

**Women's Chronic Diseases.**

Dr. More Madden sends us proofs of a small publication in which he advocates the claims of "specialists" to deal with women's diseases. He admits that what he, in common with all other medical men, calls by the barbarous name of "gynæcology," is an exceedingly unstable art. "Not only," he says, "does it vary from time to time in accordance with the progress of the sciences on which it rests, but moreover it appears to undergo at frequent intervals other changes of a purely arbitrary character. Hardly a decade passes in which the practice of gynæcology is not in this way revolutionized by some theory which is loudly heralded into existence, and having had its brief day, is hurried into the limbo of oblivion." Is it fair to take Dr. Madden exactly at his word? If it be, one plain and by no means happy conclusion is inevitable. Ladies' doctors are either excessively stupid or excessively dishonest. For our part we have no doubt that some of them are both one and the other. But, whilst we do not wish to diminish by a hair's breadth the shameful guilt of those who treat their patients dishonestly and uncandidly, we cannot but affirm the conviction that ladies are willing victims to unworthy medical arts. Plausibility in men is the god of their idolatry; and so long as a doctor can please their minds he may do what he will with their husbands' purses. But, asks a suffering woman, what am I to do if I am never well, and can get no benefit from the family doctor? We are the first to admit the full importance of that question. The answer is this: the family doctor should undoubtedly take you to a specialist. But here is where the important decision is to be made. Who is to be the specialist? That is the question! What do you want from him? do you want plausibility, or do you want honesty? Do you want scientific capacity, or do you want snake-like cunning? There is nothing more in this difficult subject than that. Ladies' specialists may be found by the score. Some few of these are known to be both perfectly honest and perfectly capable. One or other of the specialists whose moral and professional character has been established by years of worthy conduct should be selected, and his judgment should be abided by. No specialty lends itself so much to knavery as gynæcology. But inasmuch as there are some gynæcologists whose character is above suspicion, no lady sufferer from chronic disease need despair of help. If she avoids those of doubtful reputation, and selects one who has been tried and proved, she cannot go wrong.

